# NOVEL APPROACHES AND OUTCOMES TO INVESTIGATE RECOVERY AND MOBILITY IN HIP FRACTURE PATIENTS

**DOI:** 10.1093/geroni/igad104.1331

**Published:** 2023-12-21

**Authors:** Carl-Philipp Jansen

## Abstract

Hip fracture is the most frequent non-intentional injury of older persons leading to hospital admission in North America and Europe. Failure to fully recover and regain pre-fracture function and mobility is considered the single most important disability symptom experienced by hip fracture patients; severe detrimental effects on older adults’ physical, mental, and psychosocial health are reported. Being able to move back and live independently at home is seen as one of the most important aspects of recovery. However, at present, mere survival and care home admission are still the predominant reported outcomes in the recovery and rehabilitation phase. Only limited attention has been given to outcomes such as days at home or mobility performance in everyday life. At the same time, aspects of mobility recovery grounded in patients’ experience have not been addressed appropriately so far. Against this background, this symposium brings together research endeavours from Europe and North America, presenting novel approaches to investigate and disentangle trajectories of hip fracture recovery in older adults. In the first two talks, healthy days at home are investigated as central outcome after hip fracture with two different foci: the contribution of cognitive impairment to sex-based differences in recovery, and pre- vs. post-fracture development. The last two talks investigate mobility in hip fracture patients, again with two foci: results of real-world sensor-based mobility performance are presented and compared against patient-reported outcomes, and a conceptual framework of walking was developed based on a systematic review on patients’ walking experience.

